# Suboptimal culture conditions induce more deviations in gene expression in male than female bovine blastocysts

**DOI:** 10.1186/s12864-016-2393-z

**Published:** 2016-01-22

**Authors:** Sonia Heras, Dieter I. M. De Coninck, Mario Van Poucke, Karen Goossens, Osvaldo Bogado Pascottini, Filip Van Nieuwerburgh, Dieter Deforce, Petra De Sutter, Jo L. M. R. Leroy, Alfonso Gutierrez-Adan, Luc Peelman, Ann Van Soom

**Affiliations:** Department of Reproduction, Obstetrics and Herd Health. Faculty of Veterinary Medicine, Ghent University, 9820 Merelbeke, Belgium; Laboratory of Pharmaceutical Biotechnology, Faculty of Pharmaceutical Sciences, Ghent University, 9000 Ghent, Belgium; Department of Nutrition, Genetics and Ethology - Faculty of Veterinary Medicine, Ghent University, 9820 Merelbeke, Belgium; Current address: Institute for Agricultural and Fisheries Research (ILVO), Animal Sciences Unit, Scheldeweg 68, 9090 Melle, Belgium; Department for Reproductive Medicine, Ghent University Hospital, 9000 Ghent, Belgium; Veterinary Physiology and Biochemistry, Department of Veterinary Sciences, University of Antwerp, 2610 Wilrijk, Belgium; Dpto. Reproduccion Animal, Instituto Nacional de Investigación, y Tecnología Agraria y Alimentaria, INIA, 28040 Madrid, Spain

**Keywords:** Bovine embryos, RNA-Seq, In vivo, In vitro production, Serum, Serum-free, Sex

## Abstract

**Background:**

Since the development of in vitro embryo production in cattle, different supplements have been added to culture media to support embryo development, with serum being the most popular. However, the addition of serum during embryo culture can induce high birthweights and low viability in calves (Large Offspring Syndrome). Analysis of global gene expression in bovine embryos produced under different conditions can provide valuable information to optimize culture media for in vitro embryo production.

**Results:**

We used RNA sequencing to examine the effect of in vitro embryo production, in either serum-containing or serum-free media, on the global gene expression pattern of individual bovine blastocysts. Compared to in vivo derived embryos, embryos produced in serum-containing medium had five times more differentially expressed genes than embryos produced in serum-free conditions (1109 vs. 207). Importantly, in vitro production in the presence of serum appeared to have a different impact on the embryos according to their sex, with male embryos having three times more genes differentially expressed than their female counterparts (1283 vs. 456). On the contrary, male and female embryos produced in serum-free conditions showed the same number (191 vs. 192) of genes expressed differentially; however, only 44 of those genes were common in both comparisons. The pathways affected by in vitro production differed depending on the type of supplementation. For example, embryos produced in serum-containing conditions had a lower expression of genes related to metabolism while embryos produced in serum-free conditions showed aberrations in genes involved in lipid metabolism.

**Conclusions:**

Serum supplementation had a major impact on the gene expression pattern of embryos, with male embryos being the most affected. The transcriptome of embryos produced in serum-free conditions showed a greater resemblance to that of in vivo derived embryos, although genes involved in lipid metabolism were altered. Male embryos appeared to be most affected by suboptimal in vitro culture, i.e. in the presence of serum.

**Electronic supplementary material:**

The online version of this article (doi:10.1186/s12864-016-2393-z) contains supplementary material, which is available to authorized users.

## Background

Since the initial development of in vitro embryo production, the technique has been applied successfully to many species for clinical, commercial, and research purposes. In the early days, it was common practice to supplement culture media with serum to support embryo development in many species. But subsequently serum has been associated with fetal overgrowth in ruminants to give the so-called Large Offspring Syndrome at birth [1]. In cattle, serum supplementation is still being used in many laboratories [2–4] probably because it increases blastocyst rates and generally gives more consistent results [5]. We recently adopted a robust serum-free culture system, consisting of Synthetic Oviduct Fluid (SOF), bovine serum albumin (BSA), and insulin-transferrin-selenium (ITS), which yields comparable blastocyst rates (~40 %) to media supplemented with serum [5, 6]. However, bovine embryos produced in these serum-free conditions have a much lower hatching rate than embryos produced in serum-containing medium [5, 6]. Nevertheless, in many other aspects, the quality of the embryos produced in serum-free conditions is superior to that of embryos produced in the presence of serum. Embryos produced in serum-free conditions showed increased freezability and, after transfer, the birthweight and incidence of abnormalities of the resulting calves was in line with that of in vivo derived embryos [1, 5]. In addition, cattle embryos produced in serum-free conditions scored higher when using traditional parameters to evaluate embryo quality [5, 6]. These quality parameters are based on blastocyst development, blastocyst cell number, ratios of inner cell mass (ICM) and trophectoderm cells, apoptotic cell ratios [6], and also the gene expression pattern of a limited number of selected genes analyzed by qPCR [7, 8]. The selection of only a few genes to check embryo quality was based on the fact that the evaluation of expression of all the genes in the genome by qPCR was a daunting task. However, this no longer represents an obstacle with recently developed RNA sequencing techniques, which have proven to be a very powerful tool for evaluating and comparing the global gene expression pattern of even single cells, and therefore also of single embryos [3, 9]. This new technique also allows the study of associated pathways that may ultimately be involved in affecting embryo quality.

We hypothesized that the similarities between embryos derived in vivo and those produced in serum-free culture conditions would also translate to the global gene expression pattern and, hence, embryos produced in serum-free conditions would resemble in vivo derived embryos more than embryos produced in the presence of serum. Nevertheless, when evaluating the global gene expression pattern of embryos, their sex needs to be taken into account, since embryos of different sexes can respond differently to stress situations [10] and this might be reflected in changes in their gene expression pattern.

Therefore, the aim of the present study was to evaluate the effect that in vitro production, either in serum-containing or serum-free conditions, might have on cattle embryos by comparing their global gene expression pattern to that of the gold standard; namely, embryos derived in vivo. Also, to determine which in vitro condition produces more in vivo-like blastocysts. Additionally, we wanted to assess the impact of in vitro culture on embryos depending on their sex.

To our knowledge, this is the first study that uses RNA sequencing to evaluate the effect of in vitro embryo production, both in serum-containing and serum-free conditions, while at the same time taking embryonic sex into account. The results provide insight into the effects that different supplementations used for in vitro production may have on cattle embryos, show clearly how male and female embryos respond differently to suboptimal culture conditions, and offer valuable information on how to improve serum-free culture systems.

## Results and discussion

### General expression profile of blastocysts produced by different culture conditions

The global gene expression pattern of 24 early blastocysts was analyzed using the Illumina HiSeq 2500 system. Eight blastocysts produced in vitro in serum-containing medium and eight other blastocysts produced in vitro in serum-free medium were compared with eight blastocysts derived in vivo, each blastocyst constituting one replicate. In addition, between three and five blastocysts of each sex were represented in every culture condition, to avoid the possibility that a sex bias could interfere with the interpretation of the results, as highlighted by Bermejo-Alvarez et al. who reported that the expression of about one third of the genes of a blastocyst are influenced by the sex [2].

**Fig. 1:**
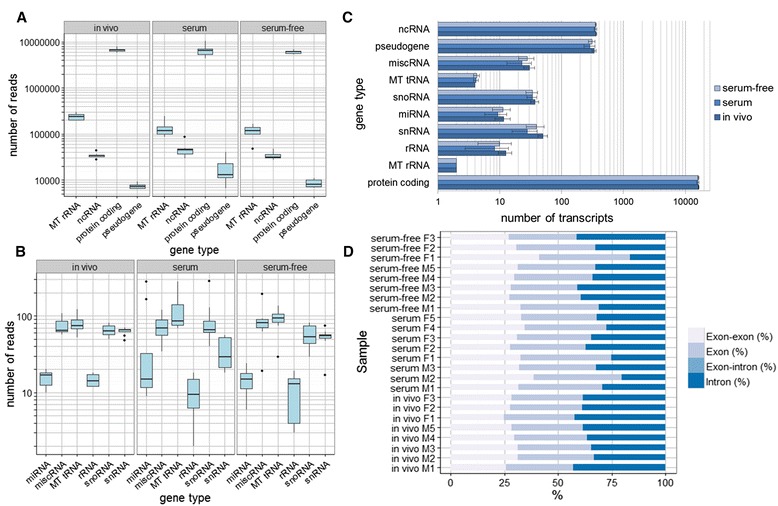
Distribution of reads and transcripts among gene types and regions. Total reads belonging to, (**a**) highly represented RNA species and, (**b**) low represented RNA species per culture condition. The total reads correspond only to total exon reads in protein-coding genes, while for the rest of the RNA species correspond to the total reads of all the gene regions. The variability of the number of reads belonging to every RNA species among the different embryos of each condition was larger in the in vitro embryos, especially in the presence of serum, compared to in vivo embryos. **c** Transcript distribution among the different RNA species in the different conditions. **d** Distribution of reads uniquely mapped among gene regions in the different blastocysts (each blastocyst constitutes a replicate)

On average, 28 million reads were generated per embryo. Of the total sequenced fragments, 51 (in vivo), 52.1 (serum-free) and 49.4 % (serum-containing) could be mapped to the Ensembl UMD 3.1 reference genome and, of these, 94 (in vivo), 93 (serum-free) and 92 % (serum-containing) were uniquely mapped to specific regions in the bovine genome (Table 1, Additional file 1: Table S1). All the uniquely mapped fragments corresponded to annotated genes and of, an average of 62 (in vivo), 67 (serum-free), and 71 % (serum-containing) mapped to annotated exons; the remainder overlapped with annotated introns (Tables 1 and Additional file 1: Table S1, Fig. 1d). Only reads uniquely mapped to exons were considered in this study since focus was placed on the expression of known annotated genes. The higher prevalence of intronic reads (~30 %) is not uncommon in bovine RNAseq experiments, when both random priming and/or oligo-dT primers are used to prepare libraries. For instance, Chitwood et al. 2013 [3] reported up to 40 % intronic reads, Graf et al. 2014 [11] around 30 %, and Huang and Khatib 2010 [12] some 20 %. Moreover, these numbers are also in line with the 30–40 % of intronic reads reported by Clontech Laboratories, manufacturers of the SMARTer Ultra Low RNA Kit–HV used for library preparation (Sara Gonzalez-Hilarion, Takara-Clontech, personal communication). A number of factors could explain the presence of intronic reads 1) the presence of pre-mRNA or unspliced RNA; 2) alternatively spliced forms, where an entire or partial intron sequence in one transcript may serve as an exon in another transcript, and 3) the presence of antisense non-coding RNAs overlapping the intronic regions; reads obtained with SMARTer Ultra Low Kits are not strand-specific so that expression coming from each strand cannot be differentiated.

**Table 1:** Summary of sequence read alignments to the reference genome

Sample	In vivo derived embryos (mean ± SD)	Serum-free produced embryos (mean ± SD)	Serum-containing produced embryos (mean ± SD)
Paired end reads	14701822.4 ± 1066098.2 × 2	12731192.8 ± 615768.7 × 2	14670296.3 ± 2890016 × 2
Total sequenced fragments	14701822.4 ± 1066098.2	12731192.8 ± 615768.7	14670296.3 ± 2890016
Total mapped fragments	7508158 ± 709020.2	6636857.8 ± 552752.8	7240822.8 ± 2076161.5
Uniquely mapped fragments	7028287.1 ± 662984.5	6174226.4 ± 523270.2	6691366.4 ± 1939025.2
Fragments mapped to annotated genes	7028287.1 ± 662984.5	6174226.4 ± 523270.2	6691366.4 ± 1939025.2
Fragments mapped to annotated exons	4353683.6 ± 619988.6	4119767.6 ± 760316.3	4730978.5 ± 1683623.3
Fragments overlapped with annotated introns	2674603.5 ± 157817.1	2054458.8 ± 430089.5	1960387.9 ± 465486.1

The numbers correspond to the mean ± standard deviation (SD) of all the replicates per condition

The distribution of total reads among different RNA species per culture condition is depicted in Fig. 1a/b. In summary, an average of 6 359 679 (96.4 %) of the total reads corresponded to protein-coding genes as a result of mRNA enrichment performed during library preparation. The next most represented RNA species was mitochondrial rRNA to which only 2.5 % of the reads corresponded. Finally, the remaining 1.1 % of the total reads was distributed among miscellaneous RNAs, including pseudogenes and different non-coding RNAs. The approximately 6 million total exon reads located in protein-coding genes corresponded to an average of 16 185 protein coding transcripts, with very similar values between the different culture conditions (Fig. 1c). The total number of genes detected in the analysis ranged from 9560 in a male embryo produced in serum-containing medium to 11 290 in a male embryo produced in serum-free medium with an average of 10 717 genes all round (Additional file 2: Table S2); this represents almost 50 % of the 22 000 protein-coding genes estimated to be present in the cattle genome [13]. The results were also in line with those of Chitwood et al. using RNA sequencing in individual blastocysts (with 11 039 genes detected) [3]. In contrast, they were lower than those found in other studies where 11 924 [9], 13 724 [11] and 17 634 genes were detected [14]. This disparity might be due to technical differences such as: 1) the sequencing depth, which usually correlates with the number of genes detected, 2) the reads per kilobase per million (RPKM) threshold used for normalization to determine when a gene is expressed and, 3) the alignment parameters that determine which reads were mapped and how non-uniquely mapping reads were dealt with.

**Fig. 2:**
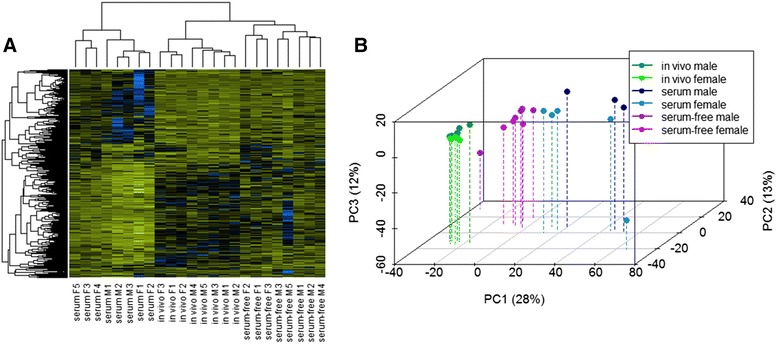
Hierarchical clustering and PCA of differentially expressed genes among the different blastocysts. **a** Heatmap including all the differentially expressed genes. The color spectrum, ranging from yellow through black to blue, represents TMM normalized expression values scaled between −4.5 and 4.5, indicating low to high expression. Two main clusters were formed, with embryos cultured in the presence of serum in one cluster and in vivo derived and serum-free embryos in the other. **b** PCA of the 24 embryos used in the study considering all the differentially expressed genes. Each dot represents one blastocyst

To verify if the embryos produced within the same condition were more similar to each other than to those produced under different conditions, a hierarchical clustering and a principal component analysis (PCA) were performed (Fig. 2). In the hierarchical clustering two clusters were formed thereby separating the embryos produced in the presence of serum from those embryos produced under the other two conditions. This cluster further divided into two minor clusters, which separated embryos derived in vivo from embryos produced in serum-free medium. Within each minor cluster the embryos were grouped by sex, except in serum conditions where female and male embryos were mixed. In the PCA, the first three principle components of the differentially expressed (DE) genes were represented and, here, blastocysts produced under the same conditions were plotted together. In the PCA, as had been already observed in the hierarchical clustering, embryos produced in serum-free conditions showed a pattern closer to in vivo derived embryos than to those produced in serum. It was also observed that the variability of gene expression within the embryos produced in serum-containing medium was greater than under the other two conditions, even though all the embryos selected for the study were early blastocysts with a score of 1 according to the International Embryo Transfer Society (IETS) guidelines. A similar observation was made by Cote et al. who compared 10 different in vitro culture conditions and in which embryos produced in the presence of serum (in both maturation and culture) presented the largest variability in the pattern of gene expression among the replicates [15].

The results of the hierarchical clustering and the PCA confirmed the rigor of the study and showed that embryos produced under the same conditions were more similar to each other than to the rest. It also gave the first indication that the global gene expression pattern of embryos produced under serum-free conditions is more similar to embryos derived in vivo than those produced in vitro in the presence of serum.

### Gene expression deviations compared to in vivo derived embryos

The number of genes differentially expressed between in vitro produced, in vivo derived embryos, with a False Discovery Ratio (FDR) corrected *p*-value of <0.05 and an absolute fold change (FC) value of ≥2, were calculated. The aim was to determine if embryos produced in serum-free conditions showed a gene expression pattern closer to that of in vivo derived embryos than embryos produced in the presence of serum (Table 2, Additional file 3: Table S3).

**Table 2:** Differentially expressed up- and down-regulated genes among the groups compared

Comparison		|FC| ≥ 0	|FC| ≥ 2	|FC| ≥ 5	|FC| ≥ 10	|FC| ≥ 20	|FC| ≥ 50	|FC| ≥ 100
All in vivo vs. all serum-containing generated	Total	2186	1109	148	51	24	8	5
	Up	1080	624	78	22	5	1	1
	Down	1106	485	70	29	19	7	4
All in vivo vs. all serum-free generated	Total	534	207	18	3	1	0	0
	Up	223	55	1	0	0	0	0
	Down	311	152	17	3	1	0	0
Male in vivo vs. male serum-containing generated	Total	1801	1283	329	136	68	25	11
	Up	919	760	236	91	46	15	6
	Down	882	523	93	45	22	10	5
Male in vivo vs. male serum-free generated	Total	311	191	28	6	1	1	1
	Up	124	59	4	1	0	0	0
	Down	187	132	24	5	1	1	1
Female in vivo vs. female serum-containing generated	Total	458	456	203	85	41	14	7
	Up	231	229	85	24	9	2	1
	Down	227	227	118	61	32	12	6
Female in vivo vs. female serum-free generated	Total	221	192	77	27	11	2	1
	Up	80	65	21	5	3	0	0
	Down	141	127	56	22	8	2	1
Male in vivo vs. female in vivo generated	Total	225	119	25	18	13	12	11
	Up	69	40	17	14	11	11	11
	Down	156	79	8	4	2	1	0
Male serum-containing vs. female serum containing generated	Total	54	54	47	35	29	17	14
	Up	17	17	15	14	12	11	11
	Down	37	37	32	21	16	6	3
Male serum-free vs. female serum-free generated	Total	54	48	19	13	9	8	8
	Up	14	14	12	10	8	8	8
	Down	40	34	7	3	1	0	0

Number of differentially expressed genes and number of up- or down regulated genes in Group 1 vs. Group 2, with an FDR corrected *p*-value of <0.05 and an absolute fold change (│FC│) ranging from ≥0 to ≥100

Considering female and male embryos together, those produced in serum-free conditions were more similar to in vivo derived embryos, having five times fewer DE genes (207) than embryos produced in serum-containing medium (1109). Remarkably, this large difference was even greater when only male embryos were compared. In male embryos produced in the presence of serum, 1283 genes were differentially expressed while the equivalent figure was only 191 in male embryos produced in serum-free conditions. When only female embryos were compared with in vivo derived embryos, the number of DE genes was drastically reduced for embryos produced in serum-containing medium, while it was maintained in embryos produced under serum-free conditions. Nevertheless, the number of DE genes in female embryos produced in serum-containing medium (456) was more than twice than that of female embryos produced in serum-free medium (192). These results indicated that serum supplementation had a greater impact on embryos than serum-free conditions, as was suggested by the clustering and PCA, with the greatest impact affecting male embryos.

In murine embryos it was reported that, after exposure of morulae to heat stress in vitro for 24 h followed by subsequent transfer to pseudopregnant recipients, only 28 % of the fetuses obtained on day 14 were males, compared to 55 % in the control group [10]. Also in humans, maternal stress during early pregnancy, or at the time of conception, led to a lower male–female ratio at term [16]. Therefore, it seems that male embryos are more susceptible to suboptimal environmental conditions than female ones and, as demonstrated in the present study, this susceptibility might be reflected in their gene expression pattern. Surprisingly, studies from previous decades repeatedly reported more male than female calves born after in vitro embryo production (using mostly media supplemented with serum) [17–19], suggesting that more male than female embryos survive after in vitro production. To explain this apparent contradiction, it has been hypothesized that, in previous days, a bias towards more male calves was introduced by the practice of selecting fast cleaving embryos for transfer [20]. Since male embryos develop faster than female embryos, more males would therefore have been transferred [20].

Not only male embryos produced in the presence of serum showed a more deviant transcriptome than their female counterparts compared to embryos derived in vivo; moreover, only 275 of their DE genes were also differentially expressed in females. Similarly, even though male and female embryos produced in serum-free conditions showed the same number of differentially expressed genes compared to embryos derived in vivo (191 and 192 respectively), only 44 of those genes were common in both sexes. This generates additional evidence to support the concept that male and female embryos respond differently to the environment. However, for the two conditions, the common DE genes of both sexes were also equally up- or down-regulated. Moreover, 38 out of the 44 common DE genes in both sexes were down-regulated in in vivo derived embryos compared to those generated in serum-free medium. In the case of embryos generated in serum-containing medium, 127 out of 275 common genes were down-regulated in vivo.

Interestingly, more DE genes (FC ≥2) were up-regulated in embryos produced in serum-free conditions compared with in vivo derived embryos, irrespective of whether all embryos, male, or female, were compared. Surprisingly, more DE genes were up-regulated in in vivo derived embryos compared to embryos produced in serum-containing medium when all and only the male embryos were considered. When all the embryos were considered, this tendency was reversed to give an FC ≥20, while when only male embryos were considered, the tendency was maintained for all FC. When female embryos were compared, the same number of DE genes were up-regulated in both groups with FC ≥2. From FC ≥5 onwards, more genes were up-regulated in embryos produced in serum-containing medium than in embryos derived in vivo (Table 2).

In a comparable study, Driver et al. reported that between embryos derived in vivo and those produced in vitro most of the DE genes were up-regulated in vivo for all the FC. Their study differed in a few important points from the present experiments. For example, they used a hybrid serum-IVM/serum-free-IVC in vitro conditions and in vivo derived embryos from non-superovulated cows, whereas we used superovulated cows. It is known that the superovulatory treatment can have an effect on the gene expression pattern of embryos recovered [21]. Moreover, no replicates were performed in the study by Driver et al., in which only one pool of in vivo derived and one pool of in vitro produced embryos were compared [14]. Therefore, whether the differences in the results between Driver et al. and the present study are due to the effect of the superovulation/in vitro production conditions on the gene expression pattern of the embryos, or to the lack of replicates in the Driver et al. study needs to be investigated further.

### Genes differentially expressed between male and female embryos

Only a few genes were differentially expressed when male and female embryos were compared under the same conditions (Table 2, Additional file 3: Table S3). In in vivo derived embryos, 119 genes were differentially expressed. Under in vitro conditions, the number of DE genes between male and female embryos decreased dramatically to less than half, with only 54 and 48 genes differentially expressed in serum-containing and serum-free medium, respectively. Of those, more genes (79, 37, and 34 respectively) were up-regulated in females than in males under all conditions.

The low number of genes found to be differentially expressed between male and female embryos contrasts with previous reports. Bermejo-Alvarez et al. showed one third (2921) of the expressed genes to be differentially expressed between male and female bovine blastocysts produced in serum-containing medium [2]. We found only 225 DE genes between sexes in in vivo derived embryos and 54 in both serum-free and serum-containing in vitro conditions (Table 2), considering in both studies FDR corrected *p*-value < 0.05 and all fold changes. These differences may be due to the large sample size used in the Bermejo-Alvarez et al. study increasing the statistical power to detect smaller differences between groups. However, when only genes with absolute fold changes of ≥2 were considered, the results of both studies became very similar. Bermejo-Alvarez et al. found that 55 genes were differentially expressed between male and female embryos produced in serum-containing medium, which corresponds almost exactly with the 54 genes that we found to be different according to sex in embryos produced in serum-containing medium. Similarly, in our serum-free medium, the number of DE genes between the sexes was 48. Only in embryos derived in vivo did we find a larger number of DE genes (119). On the other hand, Chitwood et al. found 168 genes to be differentially expressed between male and female bovine blastocysts (*p*-value <0.05 and absolute FC ≥ 2) produced in vitro in a hybrid serum-free/serum containing culture system, in which serum was added to the medium after three days of culture [3]. However, in this last study, only one female embryo was compared with four male embryos. Surprisingly, when comparing our present results with the list of DE genes provided by Bermejo-Alvarez et al., the similarities are very few. For example, out of the 85 genes with common identity to ours, 20 of them (24 %) were common with those in in vivo derived embryos, 8 (9 %) were common with embryos produced in serum-free conditions and only 2 genes (*XIST* and *BDH2*) with embryos produced in the presence of serum.

**Fig. 3:**
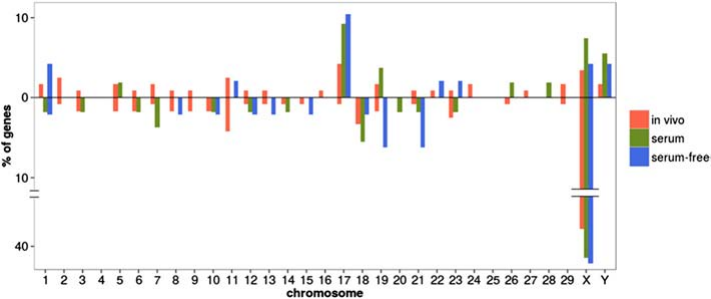
Chromosome distribution of the differentially expressed genes between male and female embryos. The bars represent the percentage of genes differentially expressed (FDR corrected p-value <0.05 and │FC│ ≥2) up- regulated (above) and down- regulated (below) in males vs. females belonging to each chromosome in each of the three conditions studied

Furthermore, the chromosomal distribution of the DE genes between the sexes was studied (Fig. 3). Here, a *χ*^2^ analysis was performed to test for significant differences in chromosome location between genes up-regulated in each sex and expressed genes. The only chromosome that displayed significant differences between DE genes up-regulated in female embryos (FDR corrected *p*-value <0.05 and absolute FC value ≥2) and expressed genes was the X chromosome under all conditions. It accounted for 54.4 in vivo, 62.2 in serum-containing, and 61.7 % in serum-free conditions of the total up-regulated DE genes in female embryos, while only 3.6 in vivo, 4 in serum-containing, and 3.8 % in serum-free embryos of the expressed transcripts were X-linked. This number of X-linked expressed transcripts is very similar to the 2.8 % reported by Bermejo-Alvarez et al. [2]. However, the percentage of X-linked genes among the up-regulated DE genes in female embryos was much higher in the present study than the level of 18.1 % noted by Bermejo-Alvarez et al. [2]. Moreover, in contrast to Bermejo-Alvarez et al., we found chromosome 17 to display significant differences between DE genes up-regulated in male embryos and expressed genes under all conditions. In fact, 12.5 for in vivo derived, 29.4 for serum-containing, and 35,7 % for serum-free of the total up-regulated DE genes in male embryos belonged to chromosome 17, while only 2.9 % of the expressed transcripts were from chromosome 17 under all conditions. Surprisingly, the X chromosome also displayed significant differences between DE genes up-regulated in male embryos produced in serum-containing medium and expressed genes, with 23.5 % of the DE genes up-regulated in males being X-linked.

### RNA-seq data validation

RNA-seq data was validated by RT-qPCR using 4 genes (PHGDH, HMGCS1, IDI1, and SFN) in 10 individual early embryos per condition, performing a total of 8 comparisons. The values obtained were normalized with 3 stable reference genes (GAPDH, YWHAZ, and SDHA). Three genes, IDI1, HMGCS1, and PHGDH, showed higher expression in embryos produced in serum-free medium compared to those derived in vivo, with averages of 4.02-,7.43-, and 3.31-fold differences, respectively, using RNA-seq and 2.94- (*p*-value <0.1), 6.14-, and 3.47- fold differences, respectively, when measured using RT-qPCR, (*p*-value <0.05; Additional file 4: Figure S1). HMGCS1 showed higher expression in embryos produced in serum-containing medium than in those derived in vivo, with an average of 1.6-fold differences when using RNA-seq and 1.67-fold differences when measuring by RT-qPCR (*p*-value <0.1). Finally, SFN showed higher expression in embryos derived in vivo compared to those produced in serum-containing medium, with an average of 10.27-fold differences using RNA-seq, and 8.56-fold differences when measuring with RT-qPCR (*p*-value <0.05). The rest of the comparisons did not show significant differences, either when analyzed by RNA-seq, or by RT-qPCR, (*p*-values in all cases <0.2). Therefore, these 4 genes showed similar patterns of mRNA abundance in RNA-seq and RT-qPCR.

### Functional analysis

The functional analysis of the differentially expressed genes between the groups was performed using the Cytoscape 3.1.1 software and considering significant only annotations with a Benjamini-Hochberg corrected *p*-value of <0.01.

**Fig. 4:**
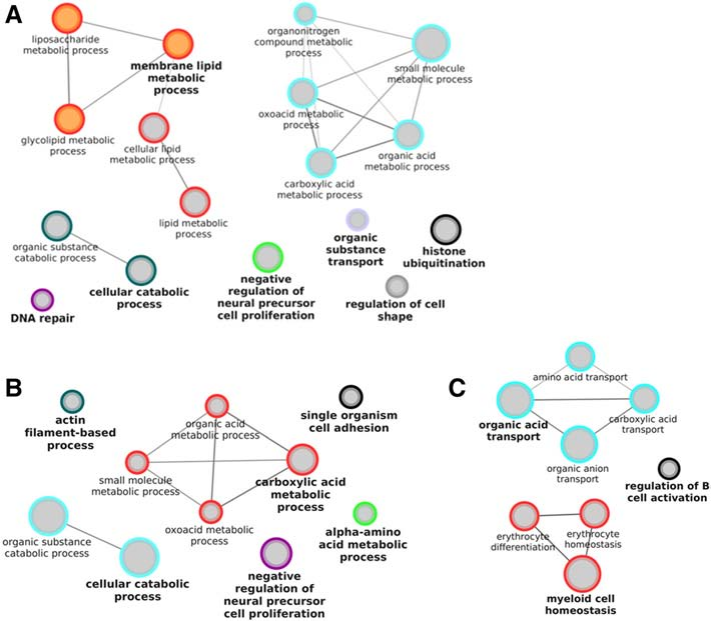
GO biological processes enriched in genes differentially expressed in in vivo vs. serum. Enriched GO biological processes with Benjamini-Hochberg corrected p-value <0.01, genes per term/pathway ≥5, Goterm levels 3–8, in the genes differentially expressed (FDR corrected p-value <0.05,│FC│ ≥2) in, (**a**) all the embryos, (**b**) only male embryos and, (**c**) only female embryos. The analysis was performed with the ClueGO 2.1.3 plugin of the Cytoscape 3.1.1. The size of the nodules represents their significance; orange nodules are only composed of genes up-regulated in vivo, grey nodules are composed of at least 5 genes up- and 5 genes down-regulated in vivo

When the gene ontology (GO) of the DE between all the embryos produced in serum-containing medium and all the embryos derived in vivo was examined, 17 biological processes, such as “lipid metabolic process” and “DNA repair” (Fig. 4), 8 molecular functions, such as “anion binding” and “actin binding,” and 21 cellular components were over-represented (Additional file 5: Table S4). Additionally, 6 KEGG pathways, including “lysosome” and “metabolic pathways” were over-represented. The vast majority of the genes included in the GO terms and KEGG pathways were up-regulated in in vivo derived embryos and only two biological processes (“DNA repair” and “histone ubiquitination”) and two cellular components (“organelle and intracellular organelle lumen”) had more genes up-regulated in embryos produced in serum-containing medium than in in vivo derived embryos. A reduced number of terms and pathways were over-represented when only male embryos were taken into account. In particular, 10 biological processes (Fig. 4), 4 molecular functions, 28 cellular components, and 3 KEGG pathways were over-represented, most of them in common with the previous comparison, but with a few differences such as “alpha-amino acid metabolic process” biological process, “enzyme binding” molecular function, “mitochondrion” cellular component, and “cysteine and methionine metabolism” KEGG pathway (Additional file 5: Table S4). When only female embryos were considered, 8 biological processes (Fig. 4), 4 molecular functions, and “mitochondrial matrix” cellular component were over-represented (Additional file 5: Table S4). Surprisingly, only “oxidoreductase activity, acting on CH-OH group of donors” molecular function was common in the comparison of all the embryos. When only male or female embryos were considered, most of the terms and all the pathways had more genes up-regulated in in vivo derived embryos.

**Fig. 5:**
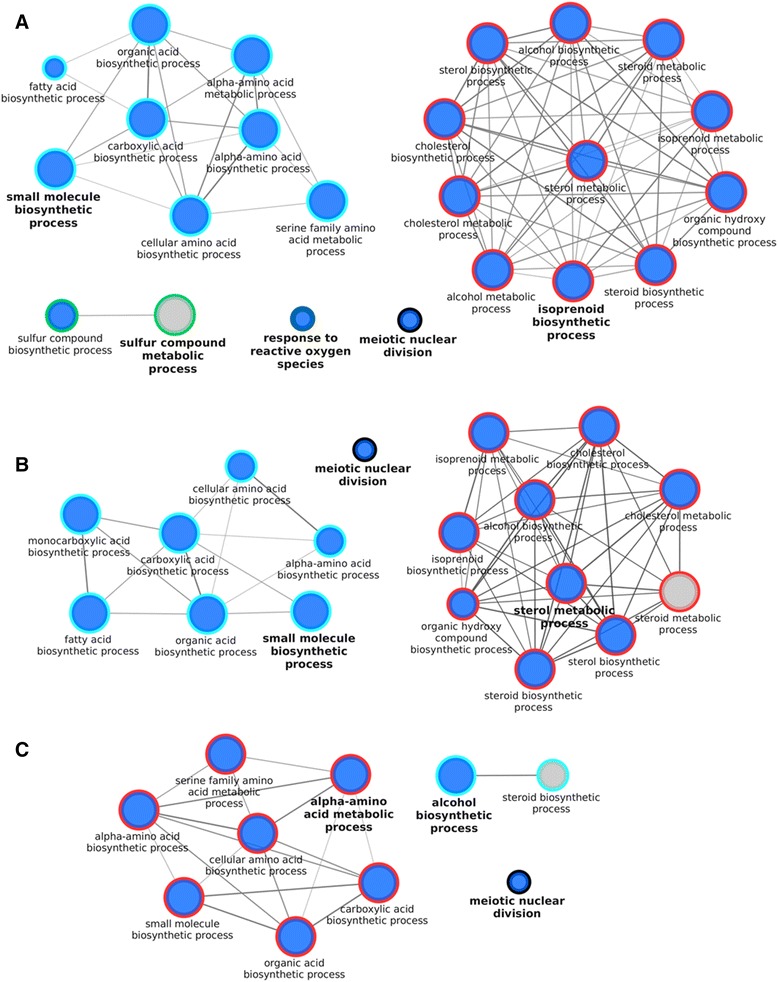
GO biological processes enriched in genes differentially expressed in the in vivo derived embryos vs. those cultured in serum-free medium. Enriched GO biological processes with a Benjamini-Hochberg corrected p-value of <0.01, genes per term/pathway ≥5, Goterm levels 3–8, in the genes differentially expressed (FDR corrected p-value <0.05,│FC│ ≥2) in, (**a**) all the embryos, (**b**) male embryos only and, (**c**) female embryos only. The analysis was done with the ClueGO 2.1.3 plugin of the Cytoscape 3.1.1. The size of the nodules represents their significance; blue nodules are composed only of genes down-regulated in vivo, grey nodules are composed of at least 5 genes up- and 5 genes down-regulated in vivo

On the other hand, the study of the gene ontology of the DE genes between all the embryos produced in serum-free medium and all the embryos derived in vivo, led to 2 molecular functions “oxidoreductase activity, acting on the CH-OH group of donors, NAD or NADP as acceptor” and “tetrapyrrole binding”, and 23 biological processes being over-represented. These biological processes were related, among other things, to cholesterol and amino acid metabolism and biosynthesis (Fig. 5). Furthermore, 3 KEGG pathways, such as the “p53 signaling pathway” and “glycine, serine, threonine metabolism” were also over-represented (Additional file 5: Table S4). If only male embryos were considered, 18 biological processes mostly common to the previous comparison (Fig. 5), two molecular functions and two KEGG pathways were over-represented (Additional file 5: Table S4). Finally, when only the female embryos were considered, 10 biological process (Fig. 5) and one KEGG pathway (“Glycine, serine, threonine metabolism”) were over-represented, all in common with the ones over-represented when all the embryos were considered (Additional file 5: Table S4). In contrast to the situation observed when comparing in vivo-derived embryos with those produced in vitro in the presence of serum, all the GO biological processes and KEGG pathways contained mostly, or even only, genes down-regulated in in vivo-derived embryos, when compared to embryos produced in serum-free medium.

In the mouse, it has been reported that during the first days of pregnancy, the mother supplies most of the cholesterol needed by the embryo [22]. Therefore, embryos derived in vivo will not have to synthetize cholesterol, in contrast to embryos produced in vitro in serum-free medium. Embryos produced in vitro in the presence of serum do not show over-representation of these pathways, indicating that serum supplementation during embryo culture provides the lipids necessary for development. In addition, the over-representation of cholesterol biosynthesis and sterol synthesis in cattle embryos produced in vitro (with serum present during maturation but the subsequent culture in serum-free conditions) compared to in vivo derived embryos was described previously by Driver et al. [14]. These results also suggest that the over expression of genes involved in lipid biosynthesis depends on the embryonic culture conditions, but is independent of the oocyte maturation conditions used, indicating that the oocytes matured in the presence of serum do not accumulate all the lipids needed for their development. Interestingly, biological processes involved in “DNA repair,” the “p53 signaling pathway” and “response to reactive oxygen species” had more genes up-regulated in embryos produced in vitro than embryos derived in vivo. This indicates that in vitro production is a source of stress for embryos regardless of culture conditions.

No functional categories were over-represented when male and female embryos were compared.

## Conclusions

Embryos produced under serum-free conditions showed gene expression patterns that were more similar to those derived in vivo than embryos produced in vitro in the presence of serum. This was true regardless of the sex of the embryos. Importantly, male embryos were most affected by suboptimal in vitro conditions, (i.e. serum supplementation) and they showed a more deviant gene expression pattern than their female counterparts. Embryos produced in the presence of serum showed reduced expression of genes related to small molecule metabolism, and an enhanced expression of genes related to DNA repair. However, embryos produced under serum-free conditions had a deviant lipid and amino acid metabolism gene expression pattern compared to in vivo derived embryos, indicating that the serum-free conditions used in this study require further optimization to fulfill the needs of the embryo during preimplantation development.

All the results of this study provide evidence for the strong and abnormal effects of adding serum to embryo culture medium. Therefore, the formulation of the culture media should move towards serum-free supplementations, even for research purposes, since experiments performed on embryos produced in the presence of serum may lead to erroneous conclusions.

## Methods

### Experimental design

In this study, the transcriptome of 16 individual bovine early blastocysts produced in vitro under two different culture conditions (eight in serum and eight in serum-free conditions) was compared to the transcriptome of eight embryos derived in vivo. Each blastocyst constituted one replicate and its sex was determined by RT-PCR prior to RNA sequencing. Blastocysts produced under each in vitro condition were only compared with the blastocysts derived in vivo. Three comparisons were made between the embryos derived in vivo, and those generated in vitro by the two different methods. First comparing all the embryos of both groups; second, comparing only the female embryos of both groups and third, comparing only the male embryos of both groups. In addition, male and female embryos of each condition were compared with each other. Consequently, a total of 9 comparisons were made (Additional file 6: Figure S2).

### In vitro embryo production

Early bovine blastocysts (*n* =16) were produced by routine in vitro methods [6]. Briefly, ovaries were collected from Holstein cows at a local slaughterhouse and processed within 2 h. Cumulus oocyte complexes were aspirated from follicles of 4–8 mm in diameter and matured in groups of 60 in 500 μL of maturation medium consisting of modified TCM-199 (GIBCO-BRL Life Technologies) supplemented, depending on the experimental design, with either 20 ng/mL EGF (Epidermal Growth Factor; Sigma E4127) and 50 μg/mL gentamycin (serum-free conditions) or 20 % heat inactivated FBS (Fetal Bovine Serum; GIBCO, Invitrogen 10108–165), 50 μg/mL gentamycin, 0.4 mM L-Glutamine and 2 mM Na-pyruvate (serum conditions) for 22 h at 38.5 °C in 5 % CO_2_-in-air. Frozen-thawed spermatozoa from the same Holstein bull of proven fertility used to obtain in vivo derived embryos (to minimize variation throughout the experiments) were passed through a discontinuous Percoll gradient (45 and 90 % (v/v); VWR International). A final sperm concentration of 1x10^6^ spermatozoa/mL was adjusted in IVF-TALP medium, consisting of bicarbonate buffered Tyrode solution supplemented with 6 mg/mL BSA (Sigma A8806) and 20 μg/mL heparin (Sigma). Matured oocytes were washed with WAS-TALP medium consisting of HEPES buffered Tyrode solution supplemented with 0.34 mg/mL BSA (Sigma A6003) before being incubated with the spermatozoa for 21 h. Presumptive zygotes were vortexed for 3 min to remove the remaining cumulus cells and spermatozoa, washed with WAS-TALP and cultured in 50 μL drops of SOF supplemented with essential and non-essential amino acids and, depending upon the experimental design, 5 % heat inactivated FBS (serum-containing conditions) or 4 mg/mL BSA (Sigma A9647) and ITS (5 μg/mL Insulin + 5 μg/mL Transferrin + 5 ng/mL Selenium; serum-free conditions). In both cases the embryos were held under mineral oil in groups of 25 at 38.5 °C in 5 % CO_2_, 5 % O_2_ and 90 % N_2_. Blastocysts were harvested, 6 and 7 days post insemination, from three independent in vitro experiments.

### In vivo embryo collection

The 8 in vivo derived blastocysts were obtained from three Holstein cows. They were superovulated with a total of 480 μg of FSH (Follicle-stimulating hormone; Stimufol) administered in eight decreasing doses every 12 h over 4 days. An injection of prostaglandin (37.5 mg; Enzaprost) was administered 48 h after the start of the superovulatory treatment. Two inseminations with frozen-thawed semen from the same Holstein bull of proven fertility used for the in vitro experiments were performed 12 h apart starting 8–12 h after the onset of the estrous behavior. Seven days after insemination, both uterine horns were flushed non-surgically for embryo recovery. The study was approved by the Ethics Committee of the Faculty of Veterinary Medicine of Gent University (EC 2012/196 and EC 2013/161).

### Embryo collection and RNA extraction

The developmental stage and quality of the blastocysts, according to IETS standards, was determined for all the embryos recovered by the same three trained individuals. Only early blastocysts of quality 1 were selected for the study. The blastocysts were washed three times in RNase-free PBS (Phosphate buffered saline; Ambion), placed individually in 2 μL of lysis buffer consisting in 5 mM DTT (DT-Dithiothreitol; Promega), 4 U/μL RNasin Plus RNase inhibitor (Promega), and 0.64 μM Igepal (Sigma) in RNase free water (Sigma) and immediately stored at −80 °C. RNA was extracted using the RNeasy micro kit (Qiagen); RNase treatment was omitted in embryos used for RNA-seq, but not in embryos used for RT-qPCR. Manufacturer′s instructions were followed with a single modification performed in the elution step, when 14 μL of RNase-free water was passed through the column twice.

### Embryo sexing

Sexing of the embryos used for RNA-seq was performed as previously described by Li et al. [23]. For sexing purposes, 2 μL of the eluted total RNA were used. RNA was reverse transcribed with the iScript™ cDNA Synthesis Kit. Next, the cDNA was amplified using the following primer pairs: DDX3Y_F, 5′-GGACGTGTAGGAAACCTTGG-3′; DDX3Y_R, 5′-GCCAGAACTGCTACTTTGTCG-3′; HPRT1_F, 5′-TGCTGAGGATTTGGAGAAGG-3′; HPRT1_R, 5′-CAACAGGTCGGCAAAGAACT-3′, and the following PCR parameters; initial denaturation at 95 °C for 3 min, 40 cycles at 95 °C for 15 s, 60 °C for 15 s and 72 °C for 30s, followed by final elongation at 72 °C for 5 min. PCR products were electrophoresed on 2 % agarose gel containing ethidium bromide and visualized under UV illumination. The *DDX3Y* gene is present on the Y chromosome, while *HPRT1* was used as the reference gene. Therefore, when one band was present, the embryo was classed as female while two bands denoted a male (Additional file 7: Figure S3).

### RNA amplification and preparation of the sequencing library

Concentration and quality of the total RNA extracted were examined using a Quant-iT RiboGreen RNA Assay kit (Life Technologies) and an RNA 6000 Pico Chip (Agilent Technologies), respectively. Subsequently, 1 ng of RNA was used to start the library preparation. First, cDNA was synthesized using the “SMARTer Ultra low input RNA for the Illumina, High Volume Kit” (Clontech) mostly following the manufacturer’s instructions. For the PCR reaction, 12 cycles were chosen. Second, the “Low Input Library Prep Kit” (Clontech) was used to prepare the libraries for sequencing. Libraries were prepared according to the manufacturer’s instructions and 4 + 6 cycles were chosen during the PCR reaction, as in the protocol. Libraries were quantified by qPCR, according to the February 2011 Illumina’s protocol “Sequencing Library qPCR Quantification protocol guide.” A high sensitivity DNA chip (Agilent Technologies) was used to control the size, distribution and quality of the libraries. Sequencing was performed by a rapid run on 2 lanes of the Illumina Hiseq-2500 sequencer using 2x100 bp paired-end reads; 12 samples were run per lane in equimolar quantities.

### Read alignment and differential gene-expression analysis

After quality trimming and trimming of 12 nucleotides from the 5′ terminal end of the reads, the latter were mapped to the *Bos taurus* UMD 3.1.75 bovine genome build (Ensembl). Specific imprinted genes of interest were added: *USP29* (NCBI Gene ID: 788661), *PEG3* (444864), *APEG3* (100169896), *MEG3* (100335527), *H19* (100126192), and *XIST* (338325), using the CLC Genomics Workbench 7.0.4 software (CLC Bio). Introns and exons were defined in the annotation. For protein-coding genes, introns and exons were distinct while the whole sequence of the ncRNAs was considered as an exon. In this study, only uniquely mapping exon reads were considered in the analysis. The quality trimming was performed based on the Phred base quality scores and a limit setting equal to 0.03. To determine if a gene was expressed, a RPKM >0.4 threshold was used for normalization (Additional file 8: Table S5). To quantify gene expression, the RNA-seq Analysis Tool from the CLC software was used, employing standard settings and mapping to gene regions only. Differentially expressed genes in the comparisons described in the experimental design were determined using the empirical analysis of the DGE tool employing standard settings. This Tool implements the ‘Exact Test’ for two-group comparisons developed by Robinson and Smyth [24] and incorporated in the EdgeR Bioconductor package [25]. The EdgeR package was used to validate the differential expression results obtained from the CLC software. To minimize false positives, within each two-group comparison, genes that did not have an exon read count of at least 15 for all the replicates in at least one of the groups under consideration were filtered out prior to differential expression analysis; this cut-off of 15 exon read counts corresponds to the inflexion point of the read frequency distribution for most samples (Additional files 9, 10 and 11: Figures S4-S6). To assess the validity of the EdgeR’s assumption of similar library distributions, boxplots of the raw read counts were rendered for the genes withheld during the previous filtering step (Additional files 12, 13 and 14: Figures S7-S9). Genes were considered to be differentially expressed if they had a Benjamini-Hochberg corrected *p*-value of <0.05 [26] and an absolute fold change of ≥2.

### Hierarchical clustering and principal component analysis

Heatmap and Principal component analyses were performed using TMM normalized read counts of genes differentially expressed (Benjamini-Hochberg corrected *p*-value <0.05, absolute FC ≥2) in at least one of the comparisons (Additional file 15: Table S6). Both analyses were performed in the R statistical software package using the heatmap and prcomp functions respectively.

### Functional annotation of genes

Ensembl gene IDs of differentially expressed genes (Benjamini-Hochberg corrected *p*-value <0.05, absolute FC ≥2) were analyzed with the ClueGO 2.1.5 plugin [27] of the Cytoscape 3.1.1 software [28] to obtain functional annotation of the genes in terms of enrichment of gene ontologies (levels 3–8) related to biological process, molecular function, and cellular component. In addition, a KEGG pathway enrichment analysis was performed. Only GO-terms or pathways that contained at least 5 of the queried genes were considered. In addition, at least 5 % of all genes associated with a GO-term or pathway needed to consist of genes in the query. Finally, results were considered statistically significant when the Benjamini-Hochberg corrected *p*-value was <0.01.

### Validation of the RNA-seq data

Quantitative real-time PCR (RT-qPCR) was used to validate the differential expression of 4 selected genes in 10 individual early blastocysts from each condition. All RT-qPCR experiments were performed according to the Minimum Information for Publication of Quantitative Real-Time PCR Experiments (MIQE) guidelines [29].

RNA from individual early blastocysts was extracted as described previously. A minus RT control was then performed with primers for GAPDH to check the removal of all the contaminating genomic DNA [30]. First-strand cDNA was generated from the total amount of RNA using the iScript cDNA synthesis kit (BioRad) which uses oligo(dT) and random hexamer primers, according to the manufacturer’s instructions. After reverse transcription, the cDNA was diluted 3-fold and used for downstream PCR. Combined with embryo sexing, cDNA quality control was performed based on Verbeke et al. [31]. For this assay, a primer pair of HPRT1 (reference gene) and a primer pair of DDX3Y (present in chromosome Y), which could amplify respectively 421 and 196 bp, were used. Only embryos that could amplify the 421 bp amplicon were included in the study. Good quality cDNA from male embryos showed two bands, one of the reference gene and one specific for the Y chromosome, while good quality cDNA from female embryos showed only the highest band, that of the reference gene. PCR reactions were performed in 10 μL reaction volumes with the following program: initial denaturation at 95 °C for 5 min, 40 cycles at 95 °C for 45 s, 64 °C for 45 s and 72 °C for 90 s, followed by final elongation at 72 °C for 5 min. PCR products were electrophoresed on 2 % agarose gel containing ethidium bromide, visualized under UV illumination and sequenced for verification.

Reference genes used for normalization (GAPDH, YWHAZ, and SDHA) were selected according to previous studies [23, 30] and confirmed by geNorm, with M-values ranging from 0.671 to 0.628, as described by Vandesompele et al. [32]. The primers pairs for the four selected genes (PHGDH, HMGCS1, IDI1, and SFN) and the specific primer annealing temperatures are given in Additional file 16: Table S7. PCR reactions were performed in a 10 μL reaction volume on a BioRad CFX 96 PCR Detection system, including 5 μL Sso Advanced SYBR Green Supermix (BioRad), 600 nM of each primer (with the exception of SFN for which 60 nM was used) and 2 μL of diluted embryo cDNA. The PCR program consisted of an initial denaturation step at 95 °C for 3 min, 40 cycles of denaturation for 5 s at 95 °C and a combined primer annealing-extension step for 30 s at the specific primer annealing temperature, during which fluorescence was measured. A melting curve was produced afterwards by heating the samples from 70 °C to 95 °C in 0.5 °C increments for 5 s while fluorescence was measured. Each reaction was run in duplicate. PCR efficiencies were calculated by a relative standard curve of 5 points with ¼ dilution, derived from cDNA of pooled bovine blastocysts. All PCR efficiencies were between 90 and 110 %, and the correlation coefficient (R^2^) between 0.990 and 1. The geometric mean of the reference genes was used to calculate the normalization factor. The mean of the duplicates and the exact PCR efficiencies were used to calculate the raw data, which, for each gene and sample was divided by the respective normalization factor to obtain a normalized value according to the method described by Hellemans et al. [33]. The normalized values were used to determine differential expression between in vivo vs. serum-containing and in vivo vs. serum-free conditions. Normality of the data was studied and a log transformation was applied when the data were not normally distributed. The normally distributed data, before or after log transformation and with homogeneity of variances, were analyzed by One-Way ANOVA combined with Hochberg Post Hoc correction. When data was not normally distributed, the non-parametric Mann–Whitney test was performed. Differences at *p* <0.05 were considered significant.

### Availability of supporting data

The data sets supporting the results of this article are available in the NCBI Gene Expression Omnibus repository (http://www.ncbi.nlm.nih.gov/geo) under accession number GSE74675.

## Additional files

Additional file 1: Table S1. Details of the sequenced reads, fragments, and their mapping to the reference genome. (PDF 204 kb) Additional file 2: Table S2. Number genes expressed in each embryo with RPKM >0.4, each embryo constituting a replicate. (PDF 250 kb) Additional file 3: Table S3. Lists of the genes differentially expressed in all the comparisons (including all the fold-changes), with their corresponding name, fold-change, *p*-value, and FDR corrected *p*-value. A positive fold-change indicates that the gene is up-regulated in Group 1 vs. Group 2. Correspondingly, a negative fold-change indicates that the gene is down-regulated in Group 1 vs. Group 2. (XLSX 423 kb) Additional file 4: Figure S1. Comparison of the differential expression of 4 genes (SFN, HMGCS1, IDI1, and PHGDH) between in vivo derived vs. serum-containing produced (top) and in vivo derived vs. serum-free produced (bottom) embryos analyzed by RNA-seq (dark grey) vs. RT-qPCR (light grey). Only comparisons that showed differences at *p*-value <0.1 are depicted. (PNG 16 kb) Additional file 5: Table S4. GO, in terms of the biological process, molecular function, and cellular component, and KEGG pathways of the genes differentially expressed (FDR corrected *p*-value <0.05, │FC│ ≥2) in all the comparisons performed. (XLSX 66 kb) Additional file 6: Figure S2. Graphic representation of the experimental design. The number of embryos present in the Figure represent the number of embryos included in the study per sex and condition. The arrows symbolize the 9 comparisons performed: red arrows represent the comparisons between female embryos; blue arrows depict the comparisons between male embryos; empty arrows represent the comparisons which included all the embryos, male and female, together; finally, black arrows are used for the comparisons between the sexes within each condition. (PNG 187 kb) Additional file 7: Figure S3. For embryo sexing, *DDX3Y*, a gene present on the Y chromosome, and *HPRT*, as reference gene, were used. The embryos that expressed only the *HPRT* transcript were considered to be female, while those that expressed the two transcripts were considered to be males. (PNG 33 kb) Additional file 8: Table S5. RPKM values of all the genes per embryo and condition. (XLSX 4083 kb) Additional file 9: Figure S4. Read frequency distribution of each embryo derived in vivo. (PNG 206 kb) Additional file 10: Figure S5. Read frequency distribution of each embryo produced in serum-containing medium. (PNG 205 kb) Additional file 11: Figure S6. Read frequency distribution of each embryo produced in serum-free medium. (PNG 206 kb) Additional file 12: Figure S7. Raw read counts boxplots after cut–off of 15 exon read counts. (PNG 230 kb) Additional file 13: Figure S8. Raw read counts boxplots after the cut–off of 15 exon read counts. (PNG 241 kb) Additional file 14: Figure S9. Raw read counts boxplots after cut–off of 15 exon read counts. (PNG 192 kb) Additional file 15: Table S6. TMM normalized counts of all the genes per embryo and condition. (XLSX 5357 kb) Additional file 16: Table S7. Primer sequence, amplicon size, and annealing temperature, and PCR efficiency of the primers of the genes used for RNA-seq validation by RT-qPCR, reference genes and genes used for the sexing and quality control assay. (PDF 180 kb)

## Abbreviations

SOF: Synthetic Oviduct Fluid
BSA: Bovine serum albumin
ITS: Insulin-transferrin-selenium
RPKM: Reads per kilobase per million
PCA: Principal component analysis
DE: Differentially expressed
FDR: False Discovery Ratio
FC: Fold change
GO: Gene ontology
EGF: Epidermal Growth Factor
FBS: Fetal Bovine Serum
FSH: Follicle-stimulating hormone
PBS: Phosphate buffered saline
DTT: DT-Dithiothreitol

## References

[1] Young LE, Sinclair KD, Wilmut I (1998). Large offspring syndrome in cattle and sheep. Rev Reprod.

[2] Bermejo-Alvarez P, Rizos D, Rath D, Lonergan P, Gutierrez-Adan A (2010). Sex determines the expression level of one third of the actively expressed genes in bovine blastocysts. Proc Natl Acad Sci U S A.

[3] Chitwood JL, Rincon G, Kaiser GG, Medrano JF, Ross PJ (2013). RNA-seq analysis of single bovine blastocysts. BMC Genomics.

[4] Corcoran D, Fair T, Park S, Rizos D, Patel OV, Smith GW (2006). Suppressed expression of genes involved in transcription and translation in in vitro compared with in vivo cultured bovine embryos. Reproduction.

[5] George F, Daniaux C, Genicot G, Verhaeghe B, Lambert P, Donnay I (2008). Set up of a serum-free culture system for bovine embryos: embryo development and quality before and after transient transfer. Theriogenology.

[6] Wydooghe E, Heras S, Dewulf J, Piepers S, Van den Abbeel E, De Sutter P (2014). Replacing serum in culture medium with albumin and insulin, transferrin and selenium is the key to successful bovine embryo development in individual culture. Reprod Fertil Dev.

[7] Market-Velker BA, Fernandes AD, Mann MR (2010). Side-by-side comparison of five commercial media systems in a mouse model: suboptimal in vitro culture interferes with imprint maintenance. Biol Reprod.

[8] Goossens K, Van Soom A, Van Poucke M, Vandaele L, Vandesompele J, Van Zeveren A (2007). Identification and expression analysis of genes associated with bovine blastocyst formation. Bmc Developmental Biology..

[9] Jiang Z, Sun J, Dong H, Luo O, Zheng X, Obergfell C (2014). Transcriptional profiles of bovine in vivo pre-implantation development. BMC Genomics.

[10] Perez-Crespo M, Ramirez MA, Fernandez-Gonzalez R, Rizos D, Lonergan P, Pintado B (2005). Differential sensitivity of male and female mouse embryos to oxidative induced heat-stress is mediated by glucose-6-phosphate dehydrogenase gene expression. Mol Reprod Dev.

[11] Graf A, Krebs S, Zakhartchenko V, Schwalb B, Blum H, Wolf E (2014). Fine mapping of genome activation in bovine embryos by RNA sequencing. Proc Natl Acad Sci U S A.

[12] Huang W, Khatib H (2010). Comparison of transcriptomic landscapes of bovine embryos using RNA-Seq. BMC Genomics.

[13] Elsik CG, Tellam RL, Worley KC, Gibbs RA, Muzny DM, Weinstock GM (2009). The genome sequence of taurine cattle: a window to ruminant biology and evolution. Science.

[14] Driver AM, Penagaricano F, Huang W, Ahmad KR, Hackbart KS, Wiltbank MC (2012). RNA-Seq analysis uncovers transcriptomic variations between morphologically similar in vivo- and in vitro-derived bovine blastocysts. BMC Genomics.

[15] Cote I, Vigneault C, Laflamme I, Laquerre J, Fournier E, Gilbert I (2011). Comprehensive cross production system assessment of the impact of in vitro microenvironment on the expression of messengers and long non-coding RNAs in the bovine blastocyst. Reproduction.

[16] Hansen D, Moller H, Olsen J (1999). Severe periconceptional life events and the sex ratio in offspring: follow up study based on five national registers. BMJ (Clinical research ed).

[17] Massip A, Mermillod P, Van Langendonckt A, Reichenbach HD, Lonergan P, Berg U (1996). Calving outcome following transfer of embryos produced in vitro in different conditions. Anim Reprod Sci.

[18] Van Soom A, Mijten P, Van Vlaenderen I, Van den Branden J, Mahmoudzadeh AR, de Kruif A (1994). Birth of double-muscled Belgian Blue calves after transfer of in vitro produced embryos into dairy cattle. Theriogenology.

[19] Camargo LS, Freitas C, de Sa WF, de Moraes FA, Serapiao RV, Viana JH (2010). Gestation length, birth weight and offspring gender ratio of in vitro-produced Gyr (Bos indicus) cattle embryos. Anim Reprod Sci.

[20] Gutierrez-Adan A, White CR, Van Soom A, Mann MR. Why we should not select the faster embryo: lessons from mice and cattle. Reprod Fertil Dev. 2014. 10.1071/RD14216 25209560

[21] Market-Velker BA, Zhang L, Magri LS, Bonvissuto AC, Mann MR (2010). Dual effects of superovulation: loss of maternal and paternal imprinted methylation in a dose-dependent manner. Hum Mol Genet.

[22] Tint GS, Yu H, Shang Q, Xu G, Patel SB (2006). The use of the Dhcr7 knockout mouse to accurately determine the origin of fetal sterols. J Lipid Res.

[23] Li W, Goossens K, Van Poucke M, Forier K, Braeckmans K, Van Soom A, et al. High oxygen tension increases global methylation in bovine 4-cell embryos and blastocysts but does not affect general retrotransposon expression. Reprod Fertil Dev 2014. 10.1071/RD14133 25515369

[24] Robinson MD, Smyth GK (2008). Small-sample estimation of negative binomial dispersion, with applications to SAGE data. Biostatistics (Oxford, England).

[25] Robinson MD, McCarthy DJ, Smyth GK (2010). edgeR: a Bioconductor package for differential expression analysis of digital gene expression data. Bioinformatics (Oxford, England).

[26] Benjamini Y, Hochberg Y (1995). Controlling the false discovery rate: a practical and powerful approach to multiple testing. J R Stat Soc Ser B Methodol.

[27] Bindea G, Mlecnik B, Hackl H, Charoentong P, Tosolini M, Kirilovsky A (2009). ClueGO: a Cytoscape plug-in to decipher functionally grouped gene ontology and pathway annotation networks. Bioinformatics (Oxford, England).

[28] Shannon P, Markiel A, Ozier O, Baliga NS, Wang JT, Ramage D (2003). Cytoscape: a software environment for integrated models of biomolecular interaction networks. Genome Res.

[29] Bustin SA, Benes V, Garson JA, Hellemans J, Huggett J, Kubista M (2009). The MIQE guidelines: minimum information for publication of quantitative real-time PCR experiments. Clin Chem.

[30] Goossens K, Van Poucke M, Van Soom A, Vandesompele J, Van Zeveren A, Peelman LJ (2005). Selection of reference genes for quantitative real-time PCR in bovine preimplantation embryos. Bmc Developmental Biology..

[31] Verbeke J, Van Poucke M, Peelman L, De Vliegher S (2015). Differential expression of CXCR1 and commonly used reference genes in bovine milk somatic cells following experimental intramammary challenge. BMC Genet.

[32] Vandesompele J, De Preter K, Pattyn F, Poppe B, Van Roy N, De Paepe A (2002). Accurate normalization of real-time quantitative RT-PCR data by geometric averaging of multiple internal control genes. Genome Biol.

[33] Hellemans J, Mortier G, De Paepe A, Speleman F, Vandesompele J (2007). qBase relative quantification framework and software for management and automated analysis of real-time quantitative PCR data. Genome Biol.

