# Supplementary material for: Suboptimal culture conditions induce more deviations in gene expression in male than female bovine blastocysts
Source: BMC Genomics. 2016 Jan 22;17:72. doi: 10.1186/s12864-016-2393-z (PMC4724126; doi:10.1186/s12864-016-2393-z)
Supplement: Additional file 1: Table S1. — Details of the sequenced reads, fragments, and their mapping to the reference genome. (PDF 204 kb) [file 12864_2016_2393_MOESM1_ESM.pdf]

Sequencing read alignment to the reference genome of each replicate from each condition.

| **In vivo embryos** | **Female 1** | **Female 2** | **Female 3** | **Male 1** | **Male 2** | **Male 3** | **Male 4** | **Male 5** |
|---|---|---|---|---|---|---|---|---|
| Paired end reads | 16,121,606 x2 | 14,409,617 x2 | 13,843,116 x2 | 13,620,506 x2 | 16,277,723 x2 | 15,350,609 x2 | 14,148,286 x2 | 13,843,116 x2 |
| Total sequenced fragments | 16,121,606 | 14,409,617 | 13,843,116 | 13,620,506 | 16,277,723 | 15,350,609 | 14,148,286 | 13,843,116 |
| Total mapped fragments | 7,610,073 | 7,446,981 | 7,090,972 | 6,480,699 | 8,769,114 | 8,209,054 | 7,367,399 | 7,090,972 |
| Uniquely mapped fragments | 7,110,780 | 6,986,045 | 6,642,570 | 6,077,412 | 8,243,423 | 7,636,560 | 6,886,937 | 6,642,570 |
| Fragments uniquely mapped to annotated genes | 7,110,780 | 6,986,045 | 6,642,570 | 6,077,412 | 8,243,423 | 7,636,560 | 6,886,937 | 6,642,570 |
| Fragments uniquely mapped to annotated exons | 4,102,468 | 4,270,863 | 4,087,575 | 3,454,351 | 5,481,012 | 4,985,662 | 4,359,963 | 4,087,575 |
| Fragments uniquely overlapped with annotated introns | 3,008,312 | 2,715,182 | 2,554,995 | 2,623,061 | 2,762,411 | 2,650,898 | 2,526,974 | 2,554,995 |

| **Serum-free embryos** | **Female 1** | **Female 2** | **Female 3** | **Male 1** | **Male 2** | **Male 3** | **Male 4** | **Male 5** |
|---|---|---|---|---|---|---|---|---|
| Paired end reads | 12,379,410 x2 | 12,271,016 x2 | 12,396,047 x2 | 13,699,027 x2 | 13,244,900 x2 | 12,986,278 x2 | 11,817,188 x2 | 13,055,676 x2 |
| Total sequenced fragments | 12,379,410 | 12,271,016 | 12,396,047 | 13,699,027 | 13,244,900 | 12,986,278 | 11,817,188 | 13,055,676 |
| Total mapped fragments | 7,030,434 | 6,702,281 | 5,852,157 | 7,537,374 | 6,697,231 | 6,225,558 | 6,103,602 | 6,946,225 |
| Uniquely mapped fragments | 6,547,162 | 6,209,519 | 5,451,078 | 7,039,712 | 6,249,430 | 5,765,092 | 5,673,586 | 6,458,232 |
| Fragments uniquely mapped to annotated genes | 6,547,162 | 6,209,519 | 5,451,078 | 7,039,712 | 6,249,430 | 5,765,092 | 5,673,586 | 6,458,232 |
| Fragments uniquely mapped to annotated exons | 5,461,255 | 4,187,439 | 3,188,923 | 4,860,264 | 3,773,491 | 3,397,218 | 3,749,514 | 4,340,037 |
| Fragments uniquely overlapped with annotated introns | 1,085,907 | 2,022,080 | 2,262,155 | 2,179,448 | 2,475,939 | 2,367,874 | 1,924,072 | 2,118,195 |

| Serum embryos | Female 1 | Female 2 | Female 3 | Female 4 | Female 5 | Male 1 | Male 2 | Male 3 |
|---|---|---|---|---|---|---|---|---|
| Paired end reads | 11,062,303 x2 | 13,830,821 x2 | 14,529,681 x2 | 12,989,933 x2 | 13,951,124 x2 | 15,149,686 x2 | 21,084,638 x2 | 14,764,184 x2 |
| Total sequenced fragments | 11,062,303 | 13,830,821 | 14,529,681 | 12,989,933 | 13,951,124 | 15,149,686 | 21,084,638 | 14,764,184 |
| Total mapped fragments | 6,263,359 | 6,969,915 | 7,924,647 | 7,476,000 | 7,738,814 | 5,115,204 | 11,531,436 | 4,907,207 |
| Uniquely mapped fragments | 5,721,258 | 6,335,832 | 7,367,133 | 6,936,042 | 7,205,318 | 4,750,391 | 10,691,683 | 4,523,274 |
| Fragments uniquely mapped to annotated genes | 5,721,258 | 6,335,832 | 7,367,133 | 6,936,042 | 7,205,318 | 4,750,391 | 10,691,683 | 4,523,274 |
| Fragments uniquely mapped to annotated exons | 4,268,748 | 3,968,231 | 4,809,020 | 5,015,515 | 4,884,879 | 3,345,415 | 8,500,472 | 3,055,548 |
| Fragments uniquely overlapped with annotated introns | 1,452,510 | 2,367,601 | 2,558,113 | 1,920,527 | 2,320,439 | 1,404,976 | 2,191,211 | 1,467,726 |
