# Supplementary material for: Suboptimal culture conditions induce more deviations in gene expression in male than female bovine blastocysts
Source: BMC Genomics. 2016 Jan 22;17:72. doi: 10.1186/s12864-016-2393-z (PMC4724126; doi:10.1186/s12864-016-2393-z)
Supplement: Additional file 2: Table S2. — Number genes expressed in each embryo with RPKM >0.4, each embryo constituting a replicate. (PDF 250 kb) [file 12864_2016_2393_MOESM2_ESM.pdf]

Number of genes expressed per embryo in each condition with RPKM > 0.4						
**Condition**	**Replicate 1**	**Replicate 2**	**Replicate 3**	**Replicate 4**	**Replicate 5**	**Mean ± SD**
Male *in vivo*	11,022	10,750	10,414	10,370	10,654	10,642 ± 265.6
Female *in vivo*	10,919	10,785	10,467	-	-	10,724 ± 232.2
Male serum	10,276	9,560	10,168	-	-	10,001 ± 386
Female serum	10,300	11,009	11,205	11,073	11,113	10,940 ± 364.8
Male serum-free	10,733	11,115	10,451	11,290	10,776	10,873 ± 331.5
Female serum-free	10,651	11,122	10,985	-	-	10,919 ± 242.3
