# Supplementary figures and images for: Suboptimal culture conditions induce more deviations in gene expression in male than female bovine blastocysts
Source: BMC Genomics. 2016 Jan 22;17:72. doi: 10.1186/s12864-016-2393-z (PMC4724126; doi:10.1186/s12864-016-2393-z)

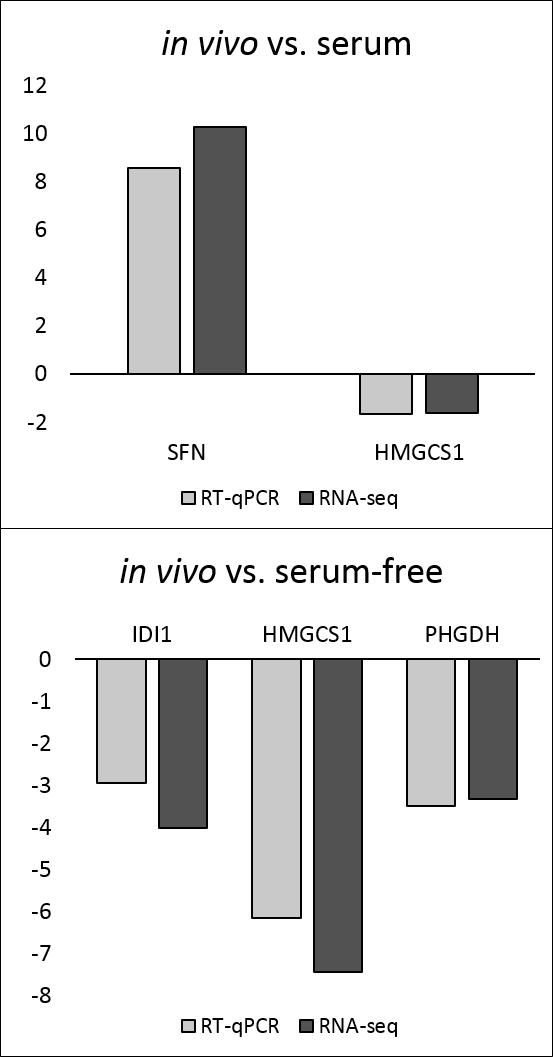

Supplement: Additional file 4: Figure S1. — Comparison of the differential expression of 4 genes (SFN, HMGCS1, IDI1, and PHGDH) between in vivo derived vs. serum-containing produced (top) and in vivo derived vs. serum-free produced (bottom) embryos analyzed by RNA-seq (dark grey) vs. RT-qPCR (light grey). Only comparisons that showed differences at p-value <0.1 are depicted. (PNG 16 kb) [file 12864_2016_2393_MOESM4_ESM.png]

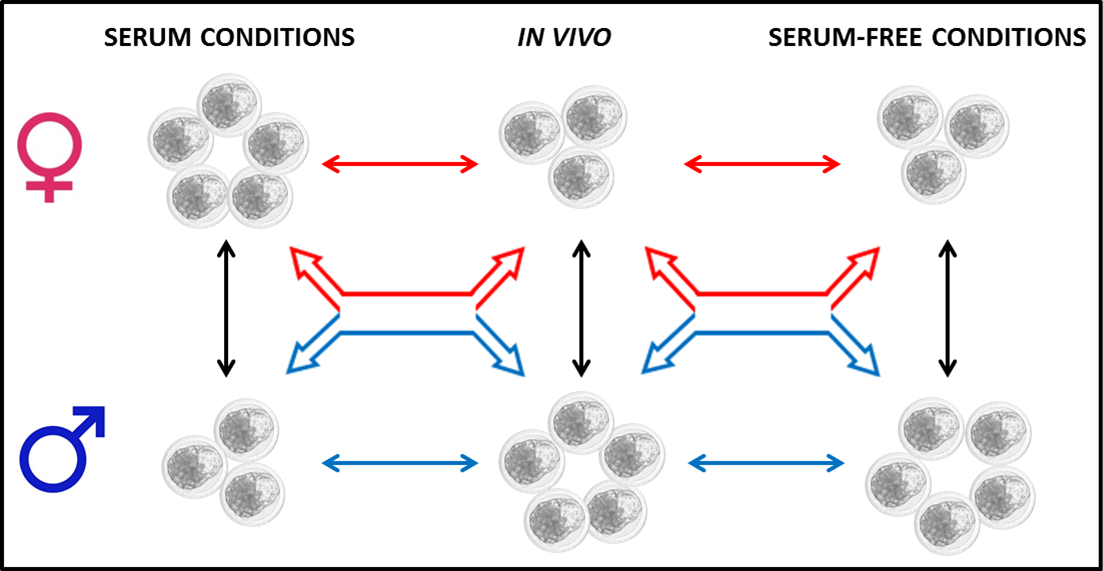

Supplement: Additional file 6: Figure S2. — Graphic representation of the experimental design. The number of embryos present in the Figure represent the number of embryos included in the study per sex and condition. The arrows symbolize the 9 comparisons performed: red arrows represent the comparisons between female embryos; blue arrows depict the comparisons between male embryos; empty arrows represent the comparisons which included all the embryos, male and female, together; finally, black arrows are used for the comparisons between the sexes within each condition. (PNG 187 kb) [file 12864_2016_2393_MOESM6_ESM.png]

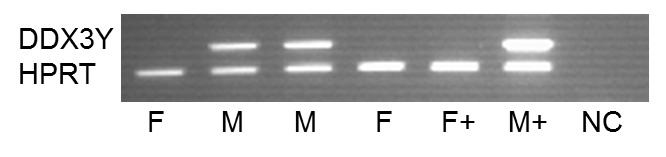

Supplement: Additional file 7: Figure S3. — For embryo sexing, DDX3Y, a gene present on the Y chromosome, and HPRT, as reference gene, were used. The embryos that expressed only the HPRT transcript were considered to be female, while those that expressed the two transcripts were considered to be males. (PNG 33 kb) [file 12864_2016_2393_MOESM7_ESM.png]

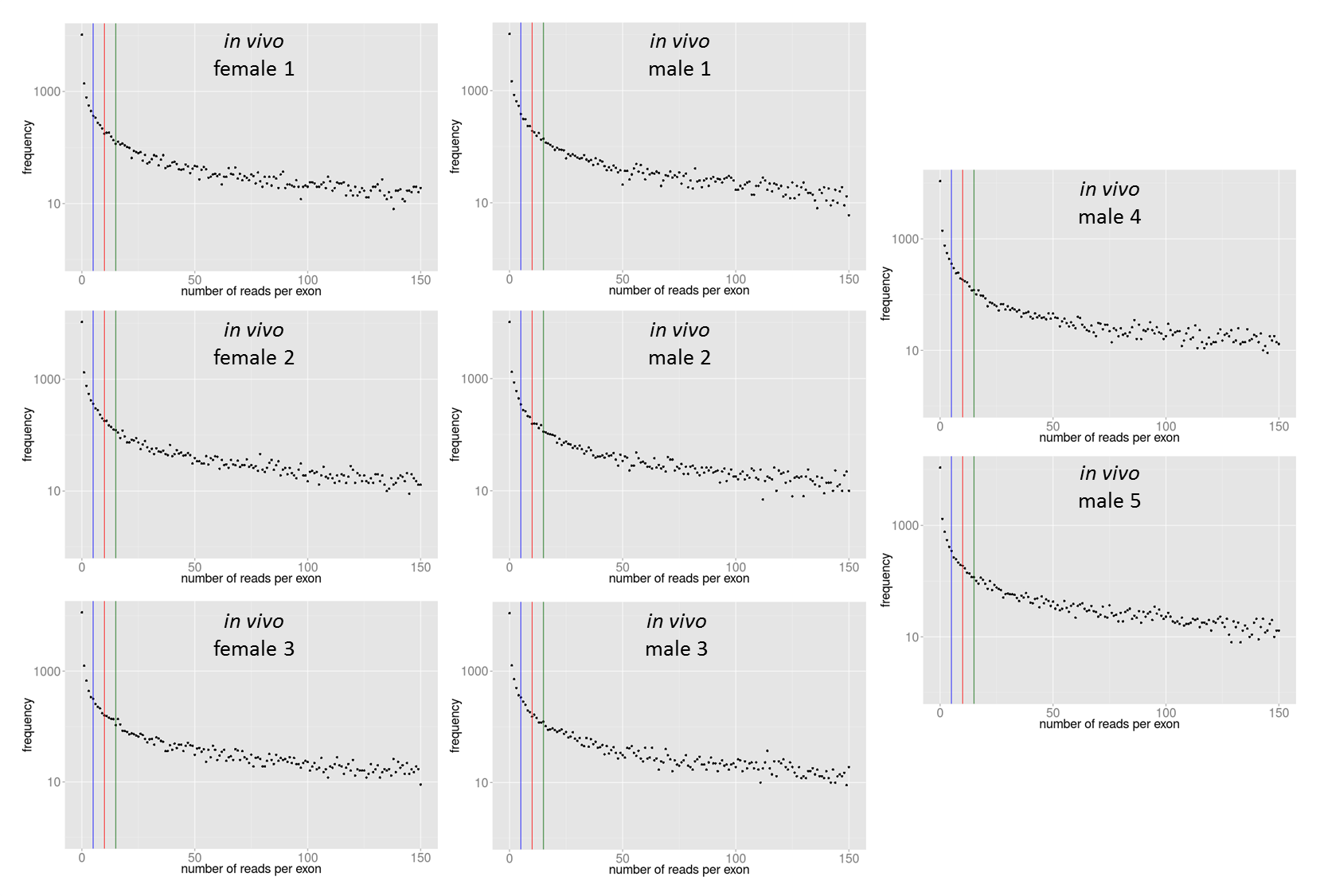

Supplement: Additional file 9: Figure S4. — Read frequency distribution of each embryo derived in vivo. (PNG 206 kb) [file 12864_2016_2393_MOESM9_ESM.png]

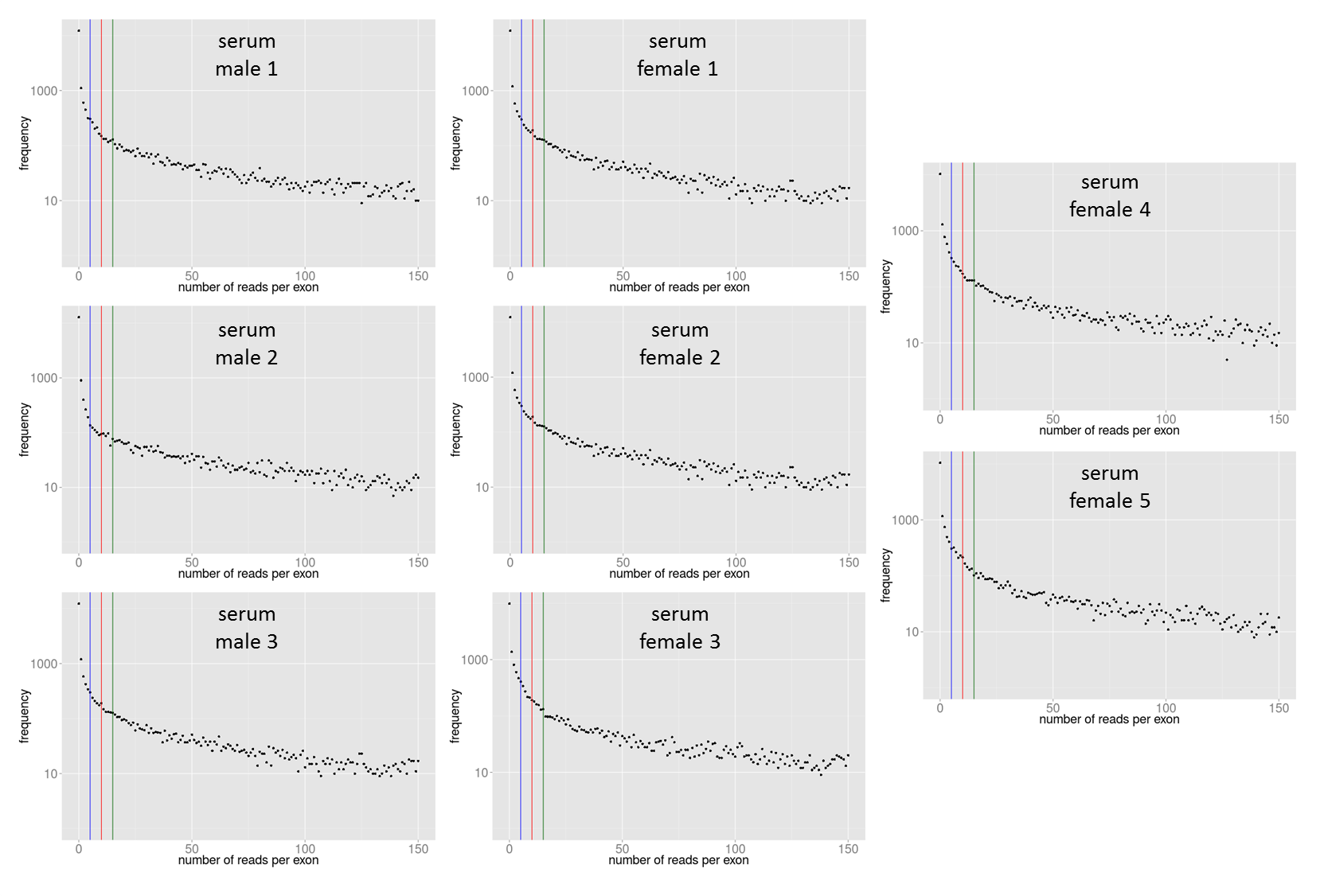

Supplement: Additional file 10: Figure S5. — Read frequency distribution of each embryo produced in serum-containing medium. (PNG 205 kb) [file 12864_2016_2393_MOESM10_ESM.png]

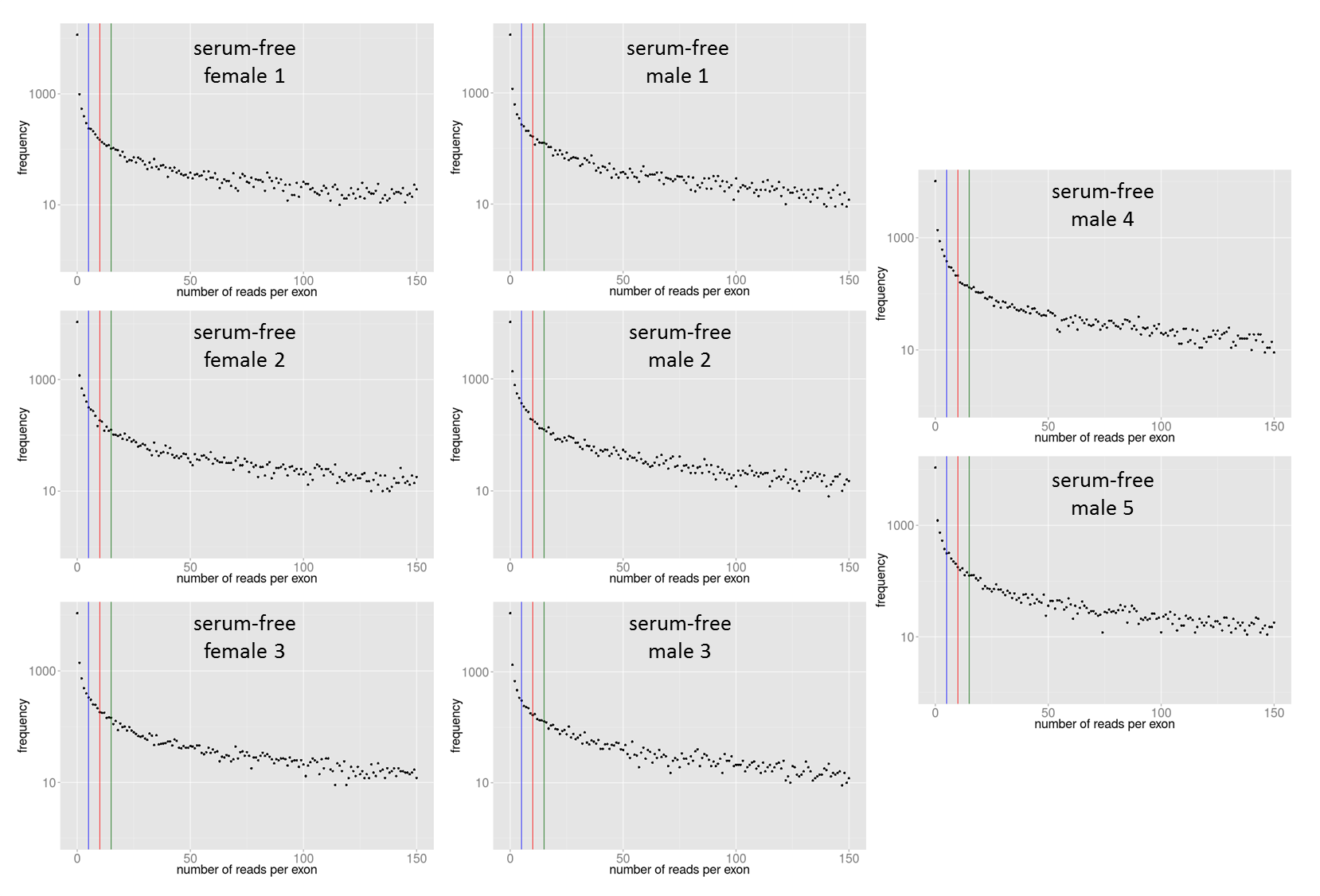

Supplement: Additional file 11: Figure S6. — Read frequency distribution of each embryo produced in serum-free medium. (PNG 206 kb) [file 12864_2016_2393_MOESM11_ESM.png]

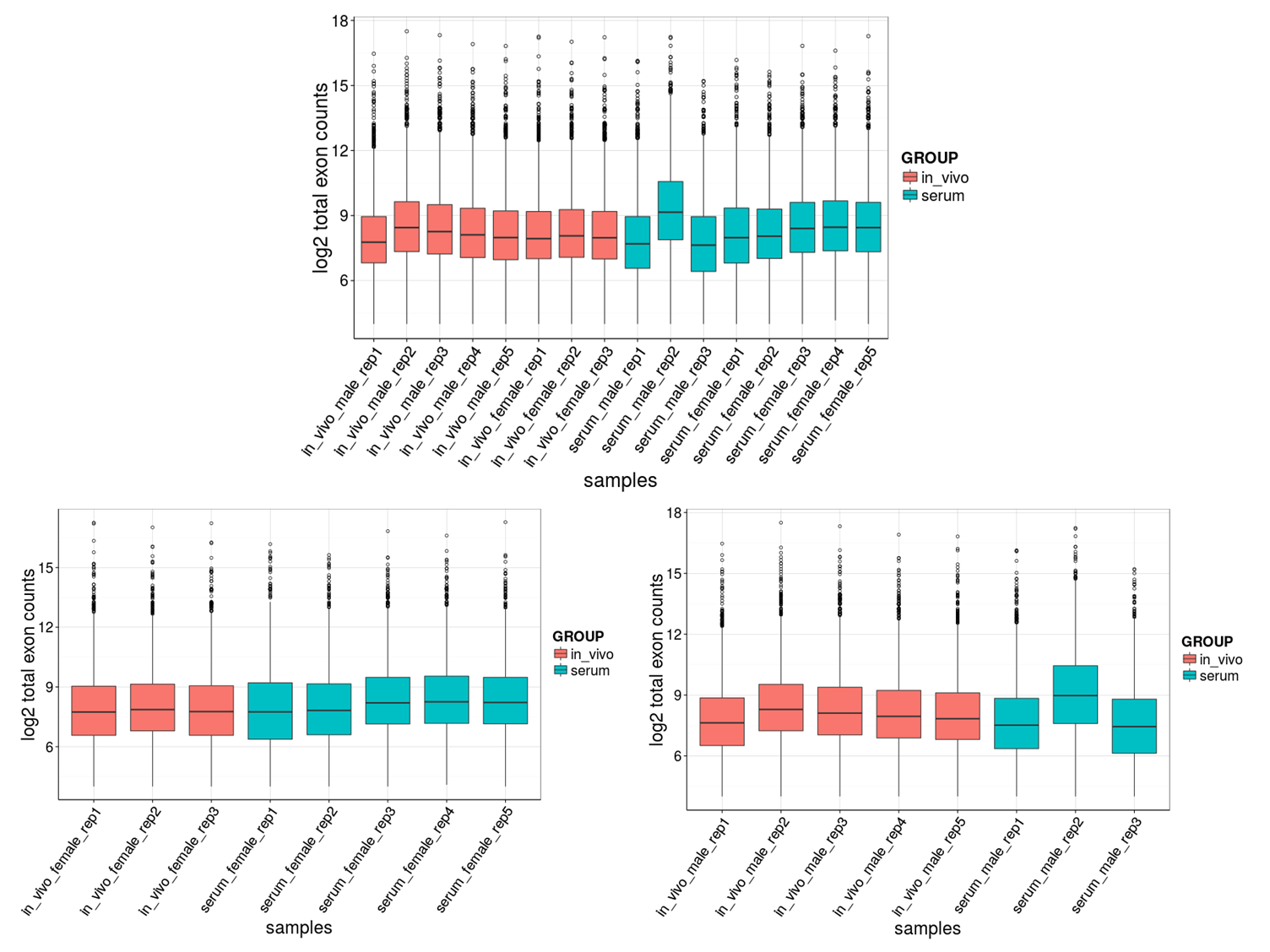

Supplement: Additional file 12: Figure S7. — Raw read counts boxplots after cut–off of 15 exon read counts. (PNG 230 kb) [file 12864_2016_2393_MOESM12_ESM.png]

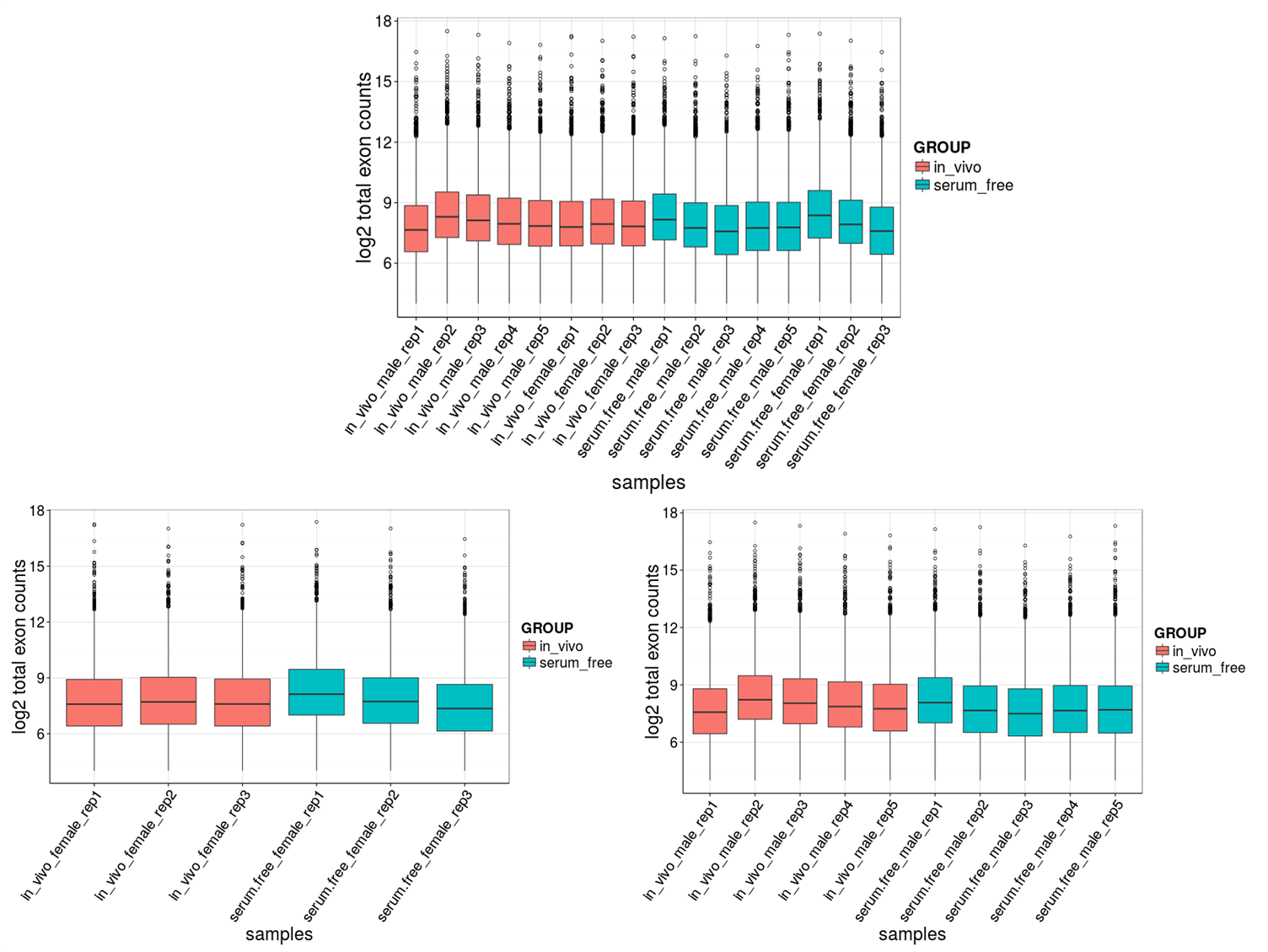

Supplement: Additional file 13: Figure S8. — Raw read counts boxplots after the cut–off of 15 exon read counts. (PNG 241 kb) [file 12864_2016_2393_MOESM13_ESM.png]

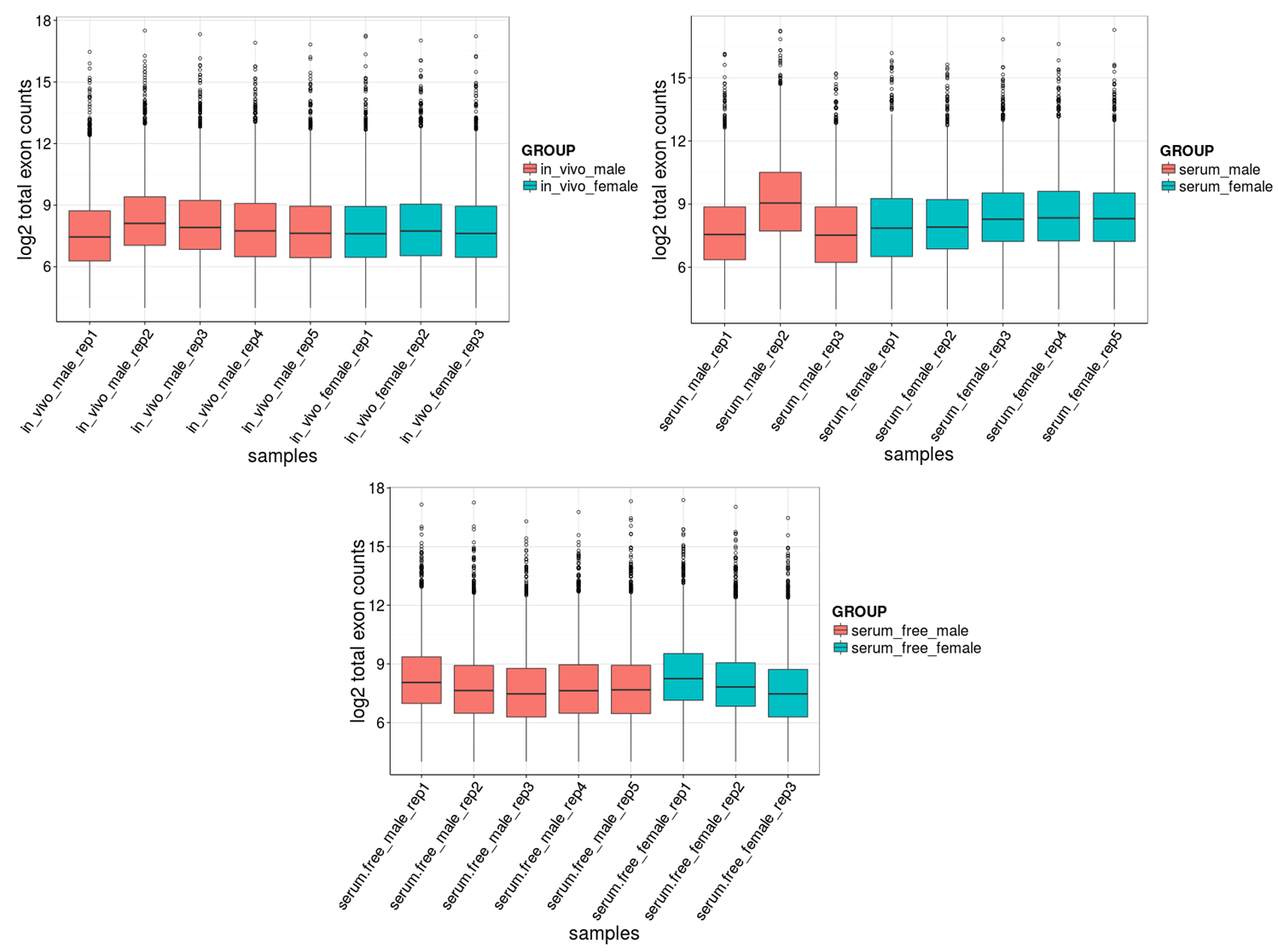

Supplement: Additional file 14: Figure S9. — Raw read counts boxplots after cut–off of 15 exon read counts. (PNG 192 kb) [file 12864_2016_2393_MOESM14_ESM.png]
