# Supplementary material for: Suboptimal culture conditions induce more deviations in gene expression in male than female bovine blastocysts
Source: BMC Genomics. 2016 Jan 22;17:72. doi: 10.1186/s12864-016-2393-z (PMC4724126; doi:10.1186/s12864-016-2393-z)
Supplement: Additional file 16: Table S7. — Primer sequence, amplicon size, and annealing temperature, and PCR efficiency of the primers of the genes used for RNA-seq validation by RT-qPCR, reference genes and genes used for the sexing and quality control assay. (PDF 180 kb) [file 12864_2016_2393_MOESM16_ESM.pdf]

Gene	Primer sequence 5´-3´	Amplicon size (bp)	Annealing Ta (°C)	PCR Efficiency
HMGCS1	Forward: CCTCAGTGCATTAGACCGCTGCT<br>Reverse: CTGAACCAGTTTACAATAGGGTGAGTGGA	142	65	100 %
IDI1	Forward: ACGCTAAGATTACCTTCCCAGGGTGT<br>Reverse: CTCTGTGCTGCTCTTCTTACTCCAATAGC	115	66	100 %
PHGDH	Forward: AGGCCGCAACCAGAAAGGGCAT<br>Reverse: TTCCGCTCCCACTTGCCATCCTT	151	67	110 %
SFN	Forward: AAAGTCGGGTCTTCTACCTGAAAATGAAG<br>Reverse: GGCATCTCCTTCTTGCTGATGTCC	145	66	100 %
GAPDH	Forward: TTCAACGGCACAGTCAAGG<br>Reverse: ACATACTCAGCACCAGCATCAC	119	62	96 %
YWHAZ	Forward: GCATCCCACAGACTATTTCC<br>Reverse: GCAAAGACAATGACAGACCA	120	60	104 %
SDHA	Forward: GCAGAACCTGATGCTTTGTG<br>Reverse: CGTAGGAGAGCGTGTGCTT	185	60	108 %
HPRT1	Forward: CCCAGCGTGGTGATTAGCGATG<br>Reverse: AAGTCTGCATTGTCTTCCCAGTGTC	421	64	
DDX3Y	Forward: AAGGCAGTTCAGGGTGGAGTTGTA<br>Reverse: CGCTCAAATCTGCCAAAGCCAGT	196	64	
